# Research on Driving Obstacle Detection Technology in Foggy Weather Based on GCANet and Feature Fusion Training

**DOI:** 10.3390/s23052822

**Published:** 2023-03-04

**Authors:** Zhaohui Liu, Shiji Zhao, Xiao Wang

**Affiliations:** 1 State Key Laboratory of Automotive Simulation and Control (ASCL), Changchun 130025, China; 2 College of Transportation, Shandong University of Science and Technology, Qingdao 266590, China

**Keywords:** traffic safety, foggy weather, GCANet defogging, edge feature, feature fusion, driving obstacle detection

## Abstract

The issues of the degradation of the visual sensor’s image quality in foggy weather and the loss of information after defogging have brought great challenges to obstacle detection during autonomous driving. Therefore, this paper proposes a method for detecting driving obstacles in foggy weather. The driving obstacle detection in foggy weather was realized by combining the GCANet defogging algorithm with the detection algorithm-based edge and convolution feature fusion training, with a full consideration of the reasonable matching between the defogging algorithm and the detection algorithm on the basis of the characteristics of obvious target edge features after GCANet defogging. Based on the YOLOv5 network, the obstacle detection model is trained using clear day images and corresponding edge feature images to realize the fusion of edge features and convolution features, and to detect driving obstacles in a foggy traffic environment. Compared with the conventional training method, the method improves the mAP by 12% and recall by 9%. In contrast to conventional detection methods, this method can better identify the image edge information after defogging, which significantly enhances detection accuracy while ensuring time efficiency. This is of great practical significance for improving the safe perception of driving obstacles under adverse weather conditions, ensuring the safety of autonomous driving.

## 1. Introduction

**Figure 1:**
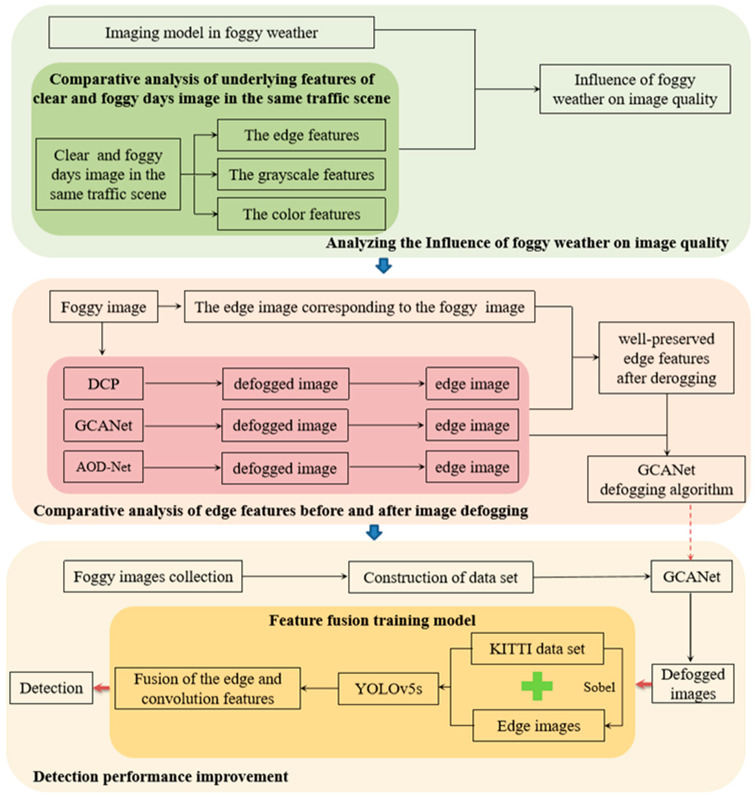
The overall technology roadmap.

In recent years, the rapid development of internet technology and data science has brought profound changes to the automobile industry. In addition, the wide application of artificial intelligence has also promoted the maturity of intelligent driving technology. Car driving is getting easier and smarter. As a development direction of future automobile driving, autonomous driving technology has been greatly developed in recent years, and it is also trying to change the face of future travel. The most basic and important aspect of automatic driving technology is environment perception. Only when the environment is accurately perceived can the safety of automatic driving be guaranteed. Therefore, the accurate perception of driving obstacles is particularly important to ensuring the safety of autonomous driving. However, as an inseparable part of the driving environment, the weather factor has always been a big challenge to improving the accuracy of automatic driving obstacle detection, which seriously affects the safety performance of automatic driving. He et al. [1] quantified the influence of fog on driving obstacle detection through simulation, and revealed the influence of the fog environment on driving obstacle detection. Among them, the problem of low visibility brought by foggy weather increases the complexity of the driving environment, and this is a difficult problem that must be faced and solved for unmanned driving. Therefore, it is necessary to start from the fog environment in order to develop a method of obstacle detection for vision sensors in bad weather driving conditions. It has practical significance as regards improving the obstacle perception performance of unmanned driving and ensuring driving safety. At present, the most commonly used driving obstacle detection methods in foggy days can be split into two categories. The first one is the method of image defogging first, followed by object detection. This method has some limitations. After defogging, the color of the object in the image will be lost, which will affect the next step of obstacle detection and the accuracy of obstacle detection. Therefore, how to solve the information loss after defogging is the challenge of the method. The second category includes the method of directly detecting obstacles by improving the target detection model without image defogging. Although this method reduces the defogging time of a single image so as to improve the detection speed of driving obstacles on foggy days, the detection accuracy of this method is low due to the visual occlusion effect, which makes it difficult to ensure the safety of automatic driving. In order to solve the problem whereby the first method has a negative effect on target detection caused by information loss after image defogging, and the second method has a low detection accuracy, this paper proposes a driving obstacle detection method using the GCA Net defogging algorithm combined with edge and convolution feature fusion training. The overall technical route is shown in Figure 1. Firstly, the reason for the low detection accuracy of driving obstacles in foggy weather is revealed by building a visual imaging model in foggy weather and analyzing low-level features of foggy images. Secondly, after comparing and analyzing the differences of the edge features between the foggy image and the defogged image, the well-preserved edge features after defogging are taken as the features fused with the convolutional features, and GCA Net is identified as the defogging algorithm of use in this paper. Finally, clear-day images from the KITTI data set and corresponding edge feature images are used to build the training set, and they are input into the YOLOv5 network for feature fusion to construct the edge and convolution feature fusion training model. Then, the images after GCANet defogging are input into the edge and convolution feature fusion training model for driving obstacle detection. The significance of this study is that it reveals the influence of foggy weather on driving obstacle detection by analyzing the differences in the low-level features of the image in clear and foggy weather, then, from the perspective of the changes in image characteristics after defogging, it is found that the target after defogging can retain good edge features, which offers new ideas for solving the problems of information loss in the image after defogging and low detection accuracy. Based on the above findings, to make full use of the edge features retained after image defogging, a training method for the fusion of edges and convolutional features is proposed. By adjusting the weight of the detection network based on fusing features, a direct connection between the target’s edge features in the image and the detection network is established, so as to further improve the detection accuracy of obstacles. Furthermore, this paper fully considers the reasonable matching between the defogging algorithm and the detection algorithm, and combines the GCANet defogging algorithm with the edge feature and convolution feature fusion training and detection algorithm to realize the detection of driving obstacles in foggy weather. Compared with other methods, this method has higher detection accuracy and provides a guarantee for the safety of automatic driving in foggy weather.

The main contributions of this paper are as follows: Firstly, by comparing and analyzing the differences of the underlying features between clear and foggy images, the influence of foggy factors on driving obstacle detection is revealed. Secondly, in order to make full use of the edge features retained in the defogged image, a training method based on the fusion of edge features and convolution features is proposed. Lastly, this paper fully considers the reasonable matching of the defogging algorithm and detection algorithm, and adopts the method of combining the GCANet defogging algorithm with edge and convolution feature fusion training, which effectively solves the problem of limited detection performance caused by information loss after image defogging.

## 2. Related Works

At present, the main method of driving obstacle detection in foggy environments is the combination of the image defogging algorithm and the target detection algorithm. The degradation of image quality in foggy weather is the most direct issue affecting target detection. Therefore, enhancing the image quality using the defogging algorithm is usually the method used to improve the detection performance.

The commonly used image defogging algorithms are generally divided into three types, based on image enhancement, image restoration and convolutional neural network. There are two types of common image enhancement algorithms; one adjusts the contrast of color in the image or performs histogram equalization on the image [2,3], and the other is an enhancement algorithm based on the Retinex principle. Wen et al. [4] verified the effect of the Retinex defogging method. As the above image enhancement algorithms do not consider the root causes of image degradation in foggy weather, the enhancement effect is limited, and the robustness is poor. In the area of image restoration, He et al. [5] proposed an image restoration method based on dark channel prior knowledge statistics, which often overestimates the fog concentration, resulting in excessive defogging and the loss of image information. Li et al. [6] selected the candidate area of the sky by combining the prior position and brightness characteristics of the image pixel points, and then reasonably estimated the atmospheric value through the pixel points of the candidate area, thus solving the problems of the color distortion and low contrast of DCP. However, the defogging effect is poor when the density distribution is uneven in foggy days, and a large number of image bottom features are lost. As for the neural network defogging method, Li et al. proposed the AOD-Net algorithm [7], which uses the convolution network to jointly estimate the global atmospheric light value and the transmission map, so as to obtain the real scene picture after defogging, which will make the reconstruction error smaller. Ma et al. [8] proposed the DbGAN defogging algorithm, which was trained using a generative countermeasure network and realized by embedding the target graph in the network generator. Fan et al. [9] proposed an image defogging algorithm based on sparse representation. The method of nonlinear stretching of the saturated component is used to improve the brightness of the image, but it is relatively simple in terms of feature extraction, resulting in local defogging and the loss of color features. Yuan et al. [10] proposed the NIN-DehazeNet defogging algorithm, based on the image of the trained model, using the estimated atmospheric light value and calculated transmission map to generate a defogged image. Yang et al. [11] proposed an auto-enhancement image defogging framework, which performs image defogging by decomposing the transmission map into density and depth. However, this method has the limitation that the depth estimation network cannot predict the depth value of the over-bright area. Yang et al. [12] proposed a predicted depth map based on a depth perception method, and provided the depth features for the denoising process in a joint framework, which realized defogging by fusing the depth features into the defogging network, but the distant fog in the image still could not be removed. Liu et al. [13] proposed a test-time training method that uses helper networks to help defogging models better adapt to the domain of interest. Song et al. [14] proposed a simple and efficient image defogging model gUNet, which uses the channel attention mechanism in the feature fusion module to extract global information, replaces pixel attention and the nonlinear activation function with a grid mechanism, and models the spatially varying transmission map. Li et al. [15] proposed a dual-scale, single-image defogging method based on neural enhancement. At the cost of the low PSNR and SSIM value of the synthesized image, a fog-free image with sharp edges and rich fine nodes can be restored for the real foggy image, but the defogging is not complete.

To sum up, the current defogging algorithm is mainly based on the theory of an atmospheric scattering model, and estimates the transmission map and atmospheric light value to obtain the real image without fog. This not only depends on prior knowledge before defogging, but also results in a loss of color, texture and other information from the image. Therefore, in view of the above problems, the GCANet (gated context aggregation network) algorithm [16] is selected for defogging processing. The key of this algorithm is that it uses smooth expansion convolution instead of expansion convolution, which not only solves the problem of grid artifacts, but also proposes a new fusion network to blend features of different levels. It does not depend on prior knowledge, and retains more image information after defogging, which significantly improves the defogging effect of the image.

In the field of computer vision, the most common target detection algorithms mainly include the two-stage detection algorithm [17,18] and the single-stage detection algorithm [19,20,21,22,23,24,25]. “Two-stage” means that the proposed candidate regions are generated first, and then the target is located and classified. Ren et al. [17] developed the traditional feature extraction method by adding the region suggestion network, thus improving the training speed of the network and realizing end-to-end training through the weight-sharing of the neural network. Qin et al. [18] proposed ThunderNet. By adding some attention modules to enlarge the receptive field using local and global features, the accuracy was effectively improved, but the detection speed was slow. “Single-stage” refers to the direct classification and positioning of targets without generating candidate regions. Common “single-stage” detection algorithms mainly include the YOLO series proposed by Redmon J, and SSD (Single-Shot MultiBox Detector) proposed by Liu W et al. [19]. Yin et al. [20] proposed the FD-SSD algorithm to solve the problem of small target detection through feature fusion and an extended convolution multibox detector. Later, Hou et al. [21] proposed a single-stage multi-target detection algorithm, which greatly improved the detection accuracy. M2Det [22] used a multi-layer pyramid structure to achieve higher accuracy. After the SSD series, the CenterNet [23], EfficientDet [24] and YOLO series of target detection algorithms stand out, and these networks gradually improve the detection speed on the basis of ensuring high detection accuracy. In 2020, Bochkovskiy et al. [25] proposed the YOLOv4 algorithm, which combined Cross-Stage-Partial-connection [26] with darknet53 [27] in the network backbone structure, and proposed the CSPDarknet53 feature extraction structure, which uses the PAN [28] structure in the neck layer, thus improving detection accuracy and speed.

In addition to the current most commonly used method of combining the defogging algorithm with the target detection algorithm, there are some methods for direct perception without defogging processing, such as detection using the illumination linear enhancement module and multi-scale depth convolution [29], foggy vehicle detection enhancing the YOLO model [30], a domain adaptation method with cycle-aware consistency [31], feature fusion training detection for sunny and foggy days [32], a method based on image style transfer [33], and a method for detecting driving obstacles in foggy days through supplementary modules of a vehicle detection system with visibility [34]. Although these methods improve the detection speed, the detection accuracy is still low. Particular in densely foggy environments, the sensing performance is poor, and it is difficult to ensure the safety of unmanned driving. After sorting out the challenges of driving obstacle detection in foggy weather and the respective methods, this paper analyzes the influence of fog on the underlying features of the image and the retention of image edge features after different defogging algorithms, to explore a scheme for improving the performance of foggy driving obstacle detection.

## 3. Foggy Image Processing

### 3.1. Imaging Model in Foggy Weather

**Figure 2:**
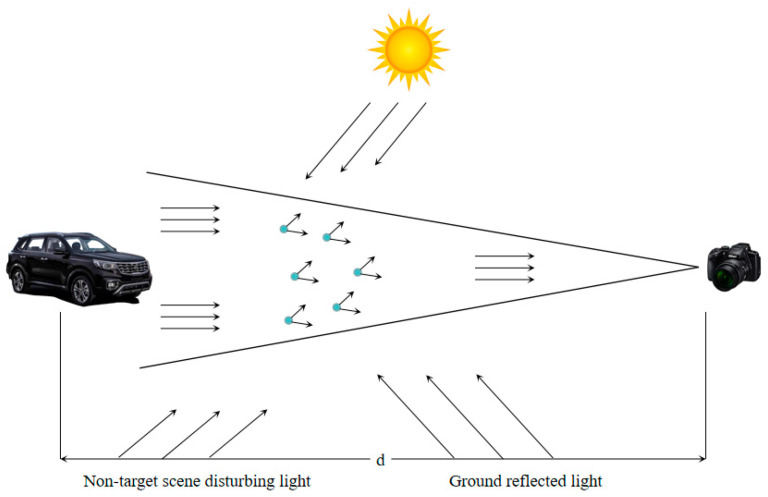
Diagram of the visual imaging model in foggy weather.

The theoretical model of atmospheric scattering proposed by McCartney [26] revealed the reasons for the loss of color information and the reduction in contrast in images in foggy weather. The visual imaging model in foggy weather consists of two parts: the incident light attenuation model and the atmospheric light reflection interference model, as shown in Figure 2. Among them, the incident light attenuation model describes the energy variation of reflected light propagating from the target scene to the collection point. Atmospheric light is mainly composed of direct light, scattered light from the sky, and reflected light from the ground. Therefore, the further the light travels, the greater the influence of atmospheric light on the fog imaging model. In relation to the theory of the atmospheric scattering model, the formulae of the visual imaging model on foggy days are as shown in Equations (1) and (2).

(1) I(u)=G(u)h(u)+V(1−h(u))

(2) $$h(u)=e^{-\alpha d(u)}$$

In Equation (1), *I*(*u*) represents the foggy image, *G*(*u*) indicates the picture after the fog is removed, and *V* refers to the global atmospheric light value, which indicates the effects of other optical paths in the atmospheric environment on the perception direction. It is usually a global constant. In Equation (2), *h*(*u*) refers to the transmittance, that is, the transmission map, which is used to describe the ability of light to penetrate suspended particles in the air on foggy days, and the value ranges from roughly 0 to 1. *α* refers to the scattering rate; *d*(*u*) represents the distance from the detection object to the vision sensors, which is the scene depth. Therefore, the image-defogging process is the process of solving the real fog-free scene *G*(*u*) using the existing foggy image *I*(*u*). That is, solving *G*(*u*) involves finding the unknown transmittance *h*(*u*) and the atmospheric light value *V*, according to *I*(*u*).

### 3.2. The Effect of Foggy Weather on Image Features

Using the visual imaging model on foggy days, it can be concluded that the impact of foggy weather on device imaging adheres to certain rules; therefore, image feature changes are also taken to be regular. In terms of foggy image characteristics, changes in the characteristics of detection objects are found, and the detection performance is then improved. Therefore, this section analyzes the changes in and differences between low-level features (such as edge features, grayscale features, and color features) of images on clear and foggy days within the same traffic scene.

#### 3.2.1. The Edge Features

**Figure 3:**
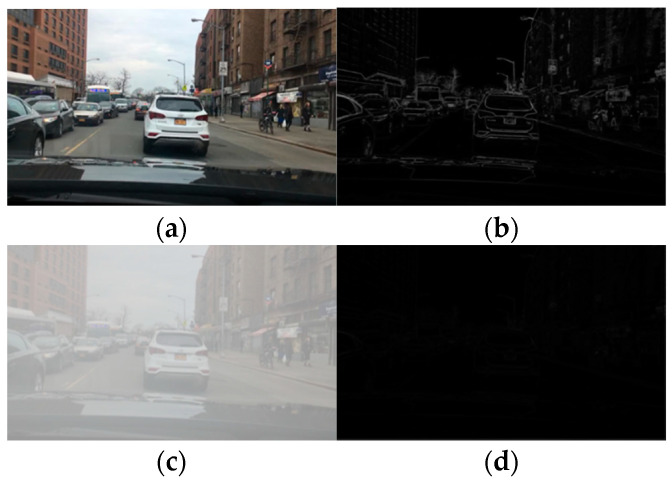
Comparison of the edge features between images of clear and foggy days for the same driving scene. (**a**) Clear day image. (**b**) Edge feature of clear day image. (**c**) Foggy image. (**d**) Edge feature of Foggy image.

The edge features of detection objects in an image refer to the discontinuity of the distribution of characteristics, such as pixel gray value and texture, or a set of pixels with step changes or ridge-like changes from the surrounding features of the image. The edge features of the target in the image communicate a lot of information about the image, and therefore the important characteristics of an image are often determined by the edge structure and characteristics. In Figure 3, for the same traffic scene, the edge features of the detection target in an image on a clear day (Figure 3a) and on a foggy day (Figure 3c) change significantly. The edge features of the detection object in the sunny day image are relatively clear, and the edge information of each object is more prominent (Figure 3b); however, the edge features in foggy weather are greatly affected, and the contour features of each detection target are relatively blurred (Figure 3d). Therefore, foggy weather will significantly weaken the edge features of an image.

#### 3.2.2. The Grayscale Features

**Figure 4:**
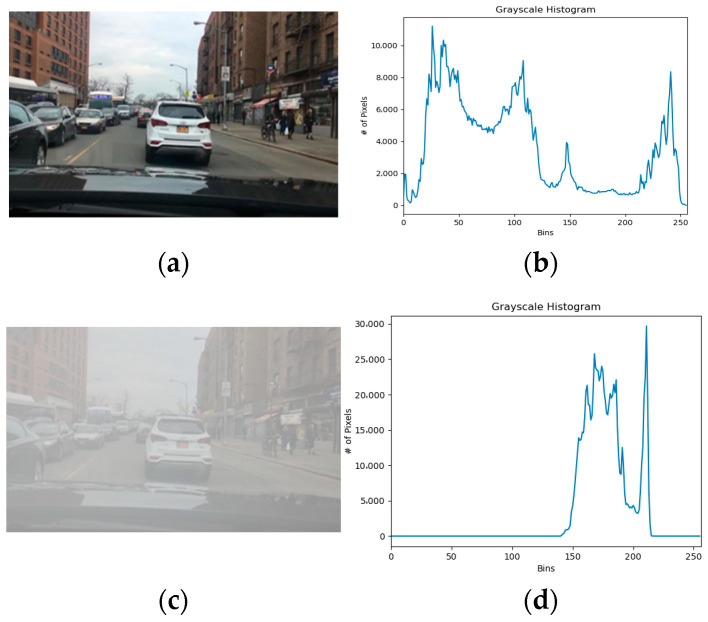
Comparison of the grayscale features between images from clear and foggy days of the same driving scene. (**a**) Clear day image. (**b**) Grayscale feature of clear day image. (**c**) Foggy image. (**d**) Grayscale feature of Foggy image.

Figure 4 shows images on clear and foggy days of the same driving scene, and the corresponding gray histogram. The horizontal axis of the histogram represents the tone, ranging from black to white, and the vertical axis represents the quantity of pixels. Comparing the images and their histograms under the two weather conditions, it can be seen that the pixel feature points in the foggy day image are significantly fewer than those in the sunny day image. In addition, the quantity of pixels in the foggy day image is highly concentrated in the tone range of 150 to 200 (see Figure 4d), which will weaken the characteristic information of the detected target in the image. Therefore, it is difficult to use foggy day images in target detection.

#### 3.2.3. The Color Features

**Figure 5:**
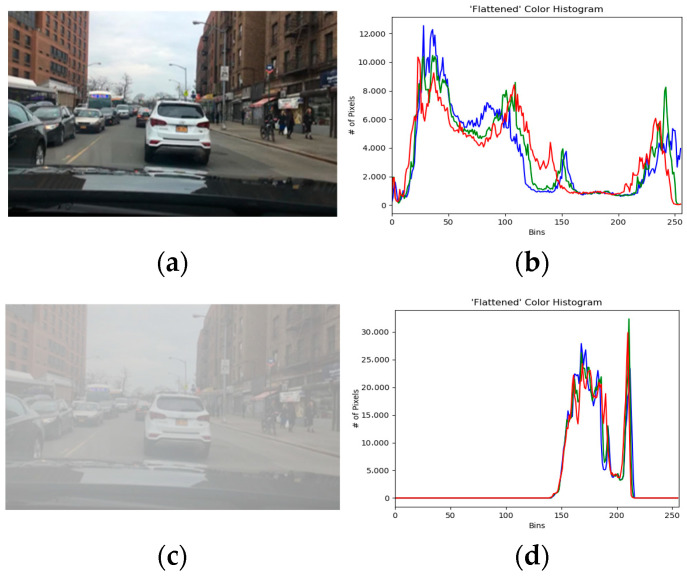
Comparison of the color features between images from clear and foggy days of the same driving scene. (**a**) Clear day image. (**b**) Color feature of clear day image. (**c**) Foggy image. (**d**) Color feature of Foggy image. In (**b**,**d**), the red curve is the red channel, the green curve is the green channel, and the blue curve is the blue channel.

Figure 5 shows images from clear and foggy days of the same driving scene, and the corresponding color histogram. The color features of an image are often represented by a color histogram. As a result of the foggy surroundings, the overall brightness level of the largest pixel in Figure 5d has moved to the right. Besides this, the quantity of pixels is greatly reduced as a whole, and several high-brightness pixels are raised. Therefore, for the same traffic scene, the global information of the image will be reduced on foggy days compared to clear days.

#### 3.2.4. The Profits and Drawbacks of the Three Underlying Characteristics

The profits and drawbacks of using the underlying features of the image in the detection of driving obstacles on foggy days are shown in Table 1, in which the edge features contain a lot of information, such as contour and position, and so the retention of edge features after defogging is analyzed.

### 3.3. Fog Image Processing and Analysis

**Figure 6:**
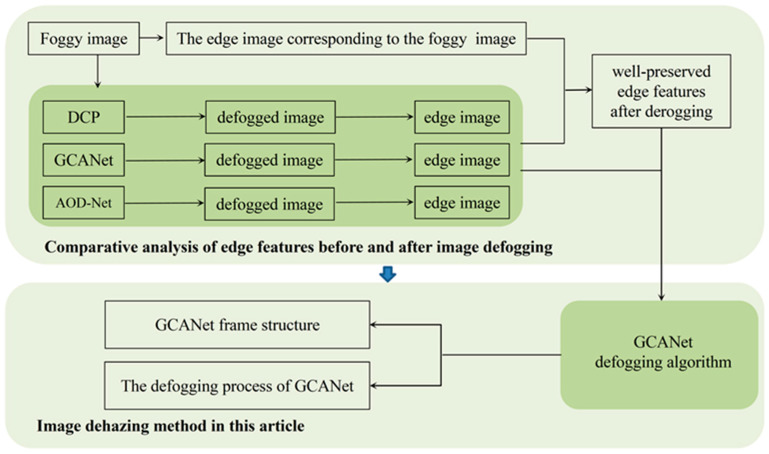
Frame diagram of image processing and analysis on foggy days.

A frame diagram of the image processing and analysis for foggy days is shown in Figure 6. Firstly, by comparing the retention of edge features after defogging using DCP, GCANet, and AOD-Net, the characteristics of edge features to be used for subsequent fusion with convolutional features are determined. Because GCANet achieves the best edge feature retention after defogging, it is used as the defogging algorithm in this paper. Finally, the GCANet defogging algorithm is introduced via the two aspects of network structure and the defogging process.

#### 3.3.1. Comparative Analysis of Edge Features before and after Image Defogging

**Figure 7:**
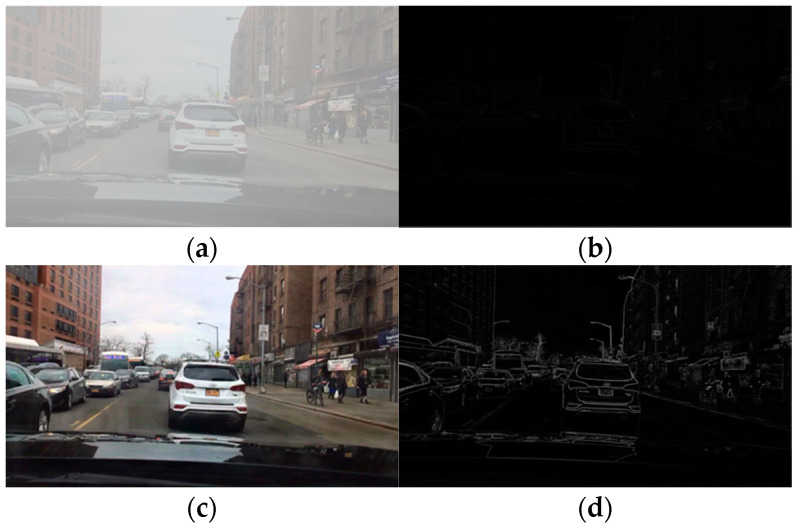

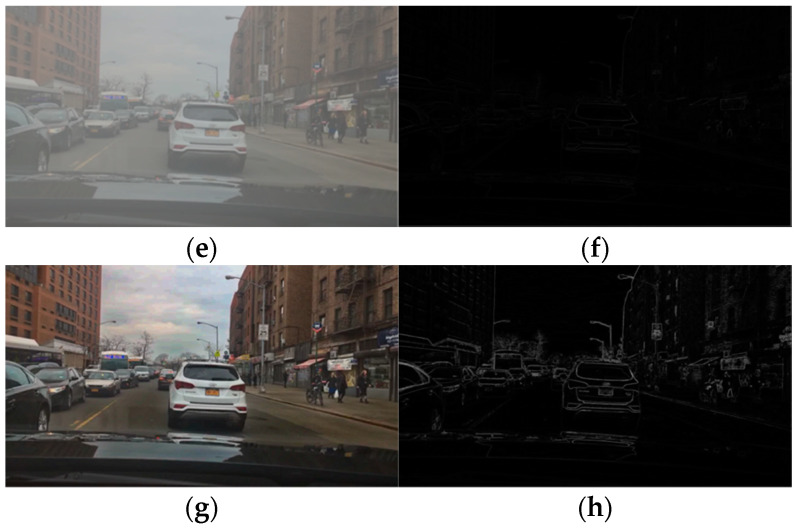
Comparison of edge features between foggy images and defogged images of the same driving scene. (**a**) Foggy image. (**b**) Edge feature of foggy image. (**c**) Image after GCANet defogging. (**d**) Edge feature map after GCANet defogging. (**e**) Image after AOD-Net defogging. (**f**) Edge feature map after AOD-Net defogging. (**g**) Image after Dark channel defogging. (**h**) Edge feature map after dark channel defogging.

Figure 7 shows a comparison of edge features between foggy day images and defogged images of the same traffic scene. It includes a foggy image, images after defogging by various methods, and the corresponding edge feature images. By comparing the edge feature images of foggy days and after defogging, it is found that the edge features are well preserved after defogging in different ways. Therefore, this paper selects edge features as the basic features to be fused with convolution features. Comparing (b), (d), (f) and (h) in Figure 7, it can be seen that the edge features are still unclear after AOD-Net defogging, and only the edge contour of the car can be vaguely detected. The edge features after DCP defogging are relatively clear, but the outlines of pedestrians are relatively blurred, and the detection object in the image after GCANet defogging retains better edge contour information, so this paper chooses GCANet as the defogging network for the processing of foggy images to retain more image information without prior knowledge.

#### 3.3.2. GCANet Defogging

Chen [16] put forwards an algorithm for end-to-end image defogging utilizing GAN (gated context aggregation network, GCANet); the algorithm does not rely on prior knowledge and uses smoothed dilated resblocks (residual block) to solve the problem of gridding artifacts. It adopts a gated fusion subnetwork to integrate features at different levels, which improves the defogging effect.

**Figure 8:**
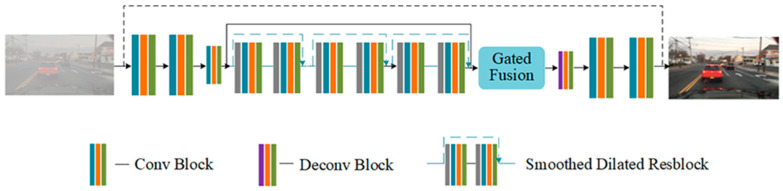
Structure of GCANet.

As shown in Figure 8, GCANet is composed of the encoder, the smoothed dilated resblock, the gated fusion subnetwork, and the decoder. The encoder comprises three consecutive convolution blocks located on the far left, and the smooth dilated residual block is a convolution block with a dotted arrow in the middle. The gated fusion subnetwork (marked in the figure) is used to fuse features at different levels, and the decoder is a deconvolution block with two convolution blocks on the far right.

The defogging process of GCANet is as follows: input the image from a foggy day, firstly encoding it into a feature map through the encoder on the left, and then the smoothed dilated Resblock and gated fusion subnetwork are used to aggregate more upper and lower information to fuse the features at multiple levels, rather than using conventional down-sampling. Finally, the object’s haze residual is obtained by decoding the augmented feature map back to the original picture space. The final defogged image is obtained by adding the haze residual to the foggy day image that was inputted. This paper does not train GCANet when using it to defog, but directly uses the pre-existing training weights on Git to defog foggy images, and then uses the edge and convolution feature fusion training model for detection.

## 4. Edge Feature and Convolution Feature Fusion Training Method

### 4.1. Training Method

**Figure 9:**
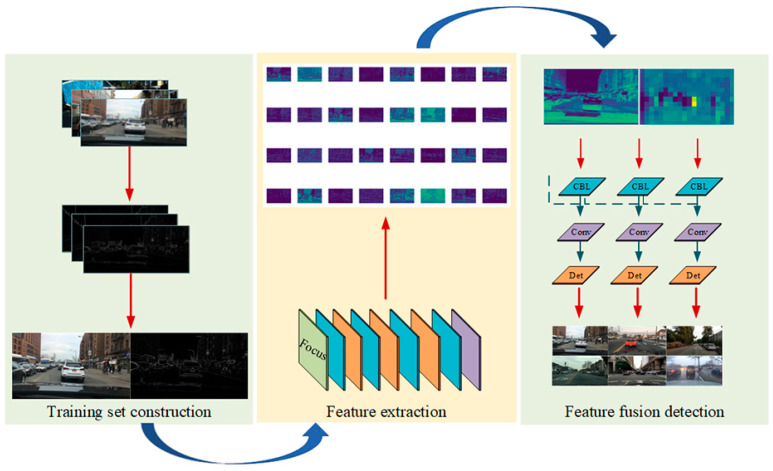
Performance improvement scheme for obstacle detection based on fusion training of edge features and convolution features.

Figure 9 depicts the edge and convolution features’ fusion training approach, which is divided into three sections: the construction of the training data set, the extraction of edge and convolution features from the obstacle, and the fusion of edge and convolution features. Firstly, the images of clear days are obtained from the KITTI data set [35], the edge features of the clear day image are extracted using the Sobel operator, and the edge feature images with abundant edge feature points and their corresponding clear day images are chosen as the training set. Then, a convolutional neural network is used to extract the convolutional features of clear day images. Eventually, through the fusion of edge features and convolution features, further convolution and tensor splicing are enacted to enhance the effect of obstacle detection in foggy weather.

### 4.2. Construction of Training Data Set

The main purpose of obstacle detection for automatic driving is to detect most of the vehicles and pedestrians, as well as non-motor vehicles such as electric vehicles and bicycles, which requires training data to meet the special requirements of the task. Therefore, the KITTI data set and the corresponding edge feature images are selected to train the network. KITTI includes real image data of various traffic scenes, and every image has up to 15 cars, 30 pedestrians, and a small number of non-motor vehicles, with different degrees of occlusion and truncation. The KITTI data set is one of the most widely used in studying the superiority of machine vision algorithms in automatic driving. In the experiment, 5985 clear day images from the KITTI data set and their corresponding edge feature images, as well as labels, were used as the training sets. We divided all the images into training sets, validation sets and test sets (8:1:1), and 749 images were used for validation. During the experiment, for the convenience of operation, the two categories of “Person_sitting” and “Pedestrian” in the KITTI data set’s labels were merged into “Pedestrian”, while the “Cyclist” category was retained, and the two categories of “Dont Care” and “Misc” were ignored.

### 4.3. Extraction of Edge Features and Convolution Features from the Obstacle

**Figure 10:**
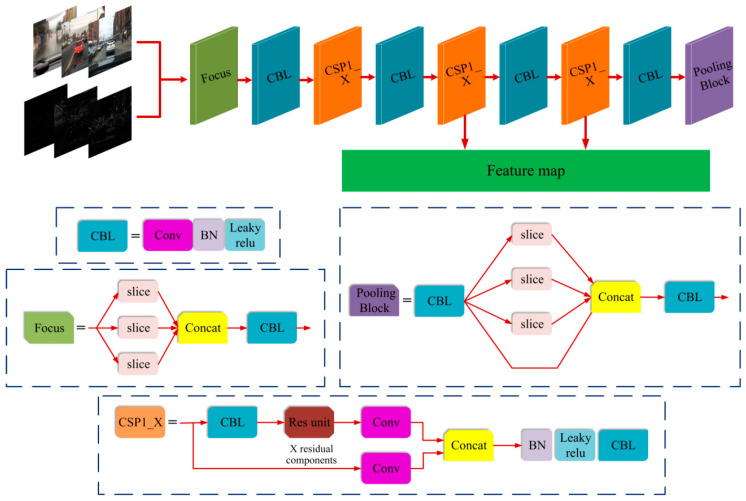
Focus and CSP1_X structure network for feature extraction of the obstacle.

To extract the edge features of the obstacles, this paper uses the clear day image from the KITTI data set, and uses the Sobel operator to extract edge features corresponding to the image in the training set. Unlike conventional object detection tasks, the detection of driving obstacles in foggy environments requires high accuracy and efficiency. Consequently, by comparing the characteristics of each feature extraction network, the Focus structure and the CSP1_X structure in YOLOv5s are selected to extract the convolutional features of obstacles. Figure 10 shows the Backbone network, composed of the Focus structure and CSP1_X structure, used for extracting feature images. After establishing the structure of the Focus and CSP1_X, nine feature layers are obtained in proper order, and the fifth and seventh feature layers are extracted by convolution processing for feature fusion and detection. CBL is an elementary structure of the feature extraction network, which adopts Batch normalization and Leaky Relu after convolution. Focus is a new structure of YOLOv5. We take the input of a 640 × 640 RGB color image as an example. When using the slice operation, it first becomes a feature map of 320 × 320 × 12, and then undergoes a convolution operation with 32 convolution kernels, and finally changes to a feature map of 320 × 320 × 32. The ninth layer in the figure is the pooling layer, which includes operations such as convolution, slicing, and Concat splicing. There are two CSP structures in YOLOv5, i.e., the CSP1_X and CSP2_X structures. We take the YOLOv5s network as an example. CSP1_X is present in the Backbone network, while CSP2_X is present in the Neck. The difference between CSP1_X and CSP2_X is that there are multiple residual blocks in CSP1_X, whereas CSP2_X is formed by replacing the residual blocks in CSP1_X with convolution blocks. CSP1_X contains multiple convolution blocks and is input into the CBL block using Batch normalization and Leaky Relu after Concat splicing.

### 4.4. Fusion of the Edge Feature and the Convolutional Feature

**Figure 11:**
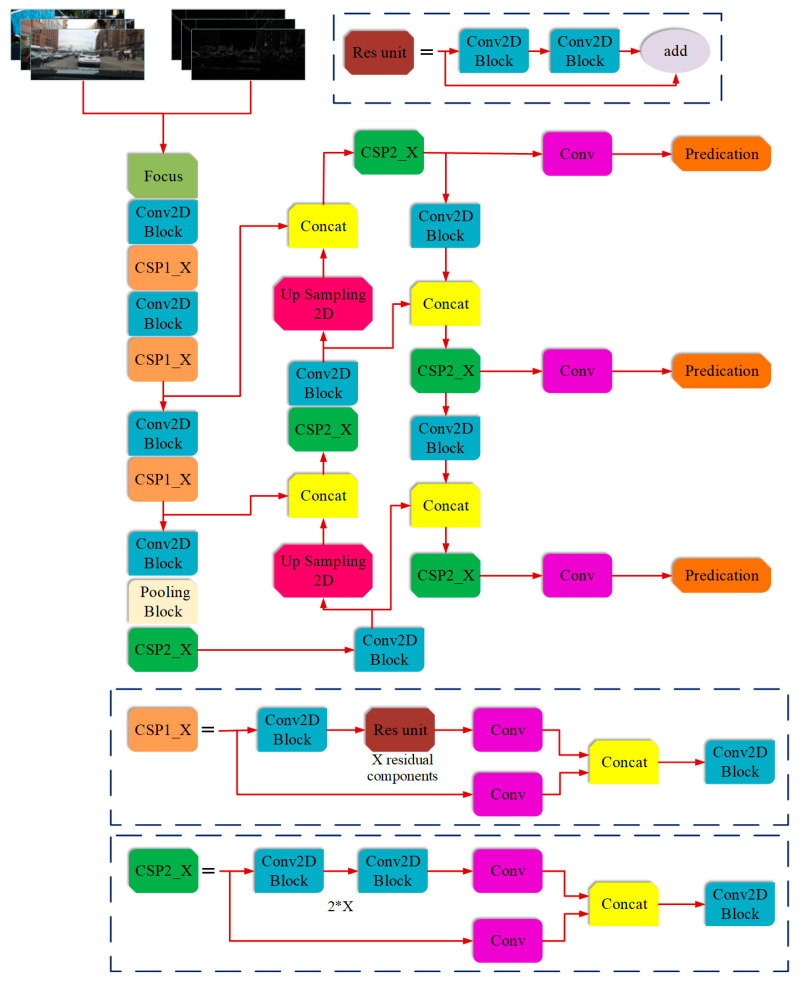
Scheme of the feature fusion and detection based on YOLOv5.

The scheme of feature fusion and detection based on YOLOv5 is shown in Figure 11. The fusion of object edge features and convolutional features not only refers to the fusion of multi-scale feature maps in the feature extraction process, but also that of edge features of obstacles in clear day images. The fused features will be used for network training to adjust model parameters. In essence, feature fusion is a form of preliminary fusion, which mainly includes add and Concat.

Add appears in the residual unit of the residual block and is reflected throughout the feature extraction process. Add is used to superimpose the feature maps so as to realize the composite of the feature maps. On the premise of not changing the dimension, it increases the amount of information of the feature image, that is, it increases the information of each feature. Concat belongs to the splicing of feature maps and exists in the CSP structure. Some output feature layers (such as the fifth layer) in Backbone are spliced with the up-sampling of the subsequent convolutional layers, thereby expanding the dimension of the feature map to realize feature fusion. Different from add, the operation of Concat realizes the information fusion of multi-scale features and the residual convolution layer, and increases the features of description for a certain kind of target in the image, rather than the features of a single target.

When using the batch of images that include both clear day images and corresponding edge feature images to train the network, add is used to add the information of convolution features and edge features to each target in the feature images of a sunny day environment, and then Concat is used to splice the target features in different environments to achieve image feature fusion. The fusion features will be utilized to train the detection network and adjust the weights of the model, so as to establish a direct connection between detected objects and edge features in the environment on a sunny day, so that the edge feature information retained through defogging can be fully used and the accuracy of detection can be improved.

## 5. Experiment Feature and Result Analysis

### 5.1. Experimental Parameters and Evaluation Indicators

#### 5.1.1. Experimental Parameters

During the training phase of the experiment, the batch size is 16, and the training is divided into 300 epochs. The initial learning rate and the final learning rate of training are 0.01 and 1, respectively. In GPU training, the graphics card is an NVDIA RTX 3060 with 12G memory, and the training image size is 640 × 640. According to the above scheme, PyTorch is used to build a training network. In total, 7481 clear day images in the KITTI data set are divided into the training set and verification set at a ratio of 8:1, of which 5984 images and their corresponding edge feature images are used for the training of the edge and convolution feature fusion, and 749 images are used as the verification set for verification. The ratio of haze-free images to edge feature images used in edge and convolution feature fusion training is 5:5. In the experiment, the edge and convolution feature fusion training is compared with the conventional training using only KITTI image data, from which four models will be obtained, namely, conventional training with KITTI data only, GCANet + training with edge images, the GCANet + conventional training model, and the GCANet + edge and convolutional feature fusion training model. The accuracy of the target detection task and classification task applied to the foggy day image test set of the four methods is compared, and the detection performance is evaluated.

#### 5.1.2. Evaluation Indicators

The most commonly used object detection evaluation indicators mainly include precision, recall and mAP (mean average precision), and their respective calculation methods (Equations (3)–(5)) are as follows:

(3) $$\mathrm{Accuracy}=\frac{\mathrm{TP}+\mathrm{TN}}{\mathrm{TP}+\mathrm{FP}+\mathrm{FN}+\mathrm{TN}}$$

(4) $$\mathrm{Precision}=\frac{\mathrm{TP}}{\mathrm{TP}+\mathrm{FP}}$$

(5) $$\mathrm{Recall}=\frac{\mathrm{TP}}{\mathrm{TP}+\mathrm{FN}}$$

TP means that the test yields a positive sample, and the actual sample is positive; TN means that the test yields a negative sample and the actual sample is positive; FP means that the test yields a positive sample and the actual sample is negative; FN means that the prediction gives a negative sample and the actual sample is positive.

It can be seen that precision reflects the adherence of the detected target to a positive sample, that is, the probability of detecting a target with a positive sample. Recall is the probability that positive sample targets in all tags will be successfully detected. When pursuing detection performance, both recall and precision should be high, but there is a certain contradiction between the two indicators. When pursuing a high recall value, negative samples may be falsely detected, so the precision value is low; when pursuing a high precision value, some positive samples may be missed. Therefore, AP (Average Precision) has been introduced as a measure of the two indicators. AP represents the area under the curve corresponding to the mean of precision when recall may take the highest value, and its calculation uses the evaluation method of the average accuracy of the difference, which is the index used to measure the accuracy of object detection. The calculation formula is Equation (6):

(6) $$\mathrm{AP}=\sum_{\alpha =0.05}^{1}P_{\alpha }$$

where AP represents the value of accuracy when recall is r. Further, mAP can be calculated according to the AP:

(7) $$\mathrm{mAP}=\frac{1}{n}\sum \mathrm{AP}$$

The mAP@0.5 used in the experiment means that the prediction is considered accurate only when the coincidence of the prediction box and label data exceeds 50%.

### 5.2. Test Set Construction

Based on Koschmieder’s law, 749 clear day images were synthesized using Matlab to obtain foggy weather images, as shown in Table 2. The synthesized foggy images were the driving images selected from the KITTI data set and the BDD100K data set [34], and the scattering rate β was set as 0.06, the global atmospheric light value A was set as 0.9, and the fog driving scene images were synthesized by adding fog to the images. Images including vehicles, pedestrians, and cyclist obstacles were selected at an interval of 20 frames. After generating the foggy image data, MakeSense was used to calibrate the target, and then the construction of the test set of foggy day images is finished. The test set for the edge and convolution feature fusion model consisted of 749 images after GCANet defogging.

### 5.3. Experimental Results

#### 5.3.1. Convergence of the Loss Function

**Figure 12:**
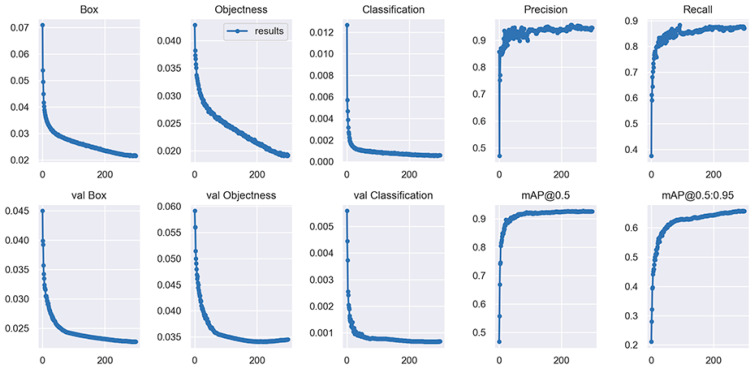
Comparison of visualization detection results of the four different methods.

The loss function reflects the difference between the predicted value and the real label in obstacle detection and classification, so it can be used to determine whether network training is effective or not. The loss function value in the fusion training of edge and convolution features is shown in Figure 12, in which Box, Objectness, and Classification correspond to object positioning loss, confidence loss, and classification loss, respectively. The cross-validation of the aforementioned three losses yielded val Box, val Objectness, and val Classification. The convergence features of the loss function show that as the Epoch number increases, losses in the network converge in the training and cross-validation sets. Therefore, the feature fusion training model proposed in this paper is effective. However the convergence of the loss function only proves the effectiveness of model training. For detection performance, the specific indicators of detection and classification need to be referred to. The changes of the precision, recall, and mAP indicators shown in Figure 11 indicate that the detection performance is gradually improved with the progression of the training.

#### 5.3.2. Visualization of Experiment Results

**Figure 13:**
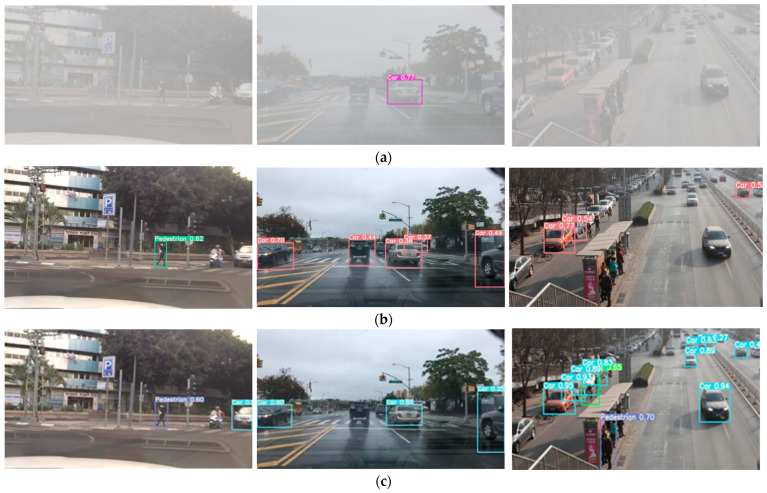

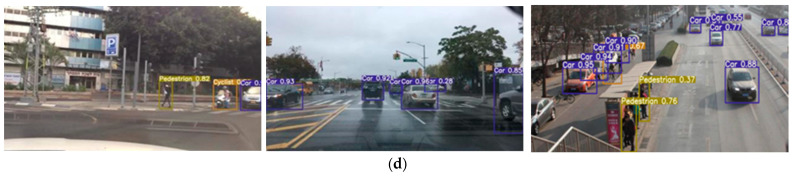
Comparison of visualization detection results of the four different methods. (**a**) Visualization results of conventional training and detection. (**b**) Visualization results of GCANet + edge images training and detection. (**c**) Visualization results of GCANet + conventional training and detection. (**d**) Visualization results of training and detection based on GCANet + the edge and convolution features fusion.

The training methods of the four models are applied to the foggy image test set for detection. Partial test results are shown in Figure 13. It can be intuitively seen that after GCANet’s defogging of foggy day images, the fusion model combining edge and convolution features has obvious advantages in improving the performance of target detection. The confidence and recall of the single target are better than those of conventional training models, especially for non-motorized vehicles and distant semi-occluded vehicles, as well as pedestrians.

#### 5.3.3. Performance of the Obstacle Detection

In order to verify the effectiveness of the edge and convolutional features fusion training method, four methods have been used to detect obstacles in foggy weather, namely, the conventional training model based only on KITTI data, the GCANet + training with edge images model, the GCANet + conventional training model, and the GCANet + edge and convolution features fusion training model. The results are shown in Table 3. Table 3 proves the effectiveness of the method used for driving obstacle detection based on GCANet image defogging combined with edge and convolution feature fusion training.

As can be seen from the data in Table 3, when only the KITTI data set is used for training and directly detecting foggy day images, the precision, recall and mAP are all low—62.5%, 48.3%, and 47.4%, respectively. It can be seen here that a foggy environment directly affects the accuracy of driving obstacle detection. Compared with the conventional training using KITTI only, when using the GCANet + edge images training model for detection, the precision, recall, and mAP were increased by 6.7%, 6.1% and 10.3%, respectively. The GCANet + conventional training model also significantly enhanced the performance of object detection, as the precision, recall, and mAP increased by 31%, 27.8%, and 35.8%, respectively; however, the recall is only 76.1%, the comprehensive index of mAP is 83.2%, and the accuracy of driving obstacle detection still shows much scope for improvement. Compared with the GCANet + conventional training detection model, the precision of the model trained by GCANet + edge and convolutional features fusion decreased by 0.8%, but the recall and mAP were greatly improved, increasing by 12% and 9.6%, respectively. The experimental results show that the training of edge and convolution feature fusion can greatly improve the applicability of vehicle-mounted visual sensors for driving obstacle detection after image restoration in foggy conditions, which is of great practical significance for the safety perception of driving obstacles under adverse weather conditions and the safety of automatic driving.

Furthermore, regarding the specific classification of driving obstacles in foggy weather, the two training models applied after GCANet defogging are compared. In car classification, the recall and mAP of the edge and convolutional feature fusion training model increased by 3.9% and 2.2%, respectively; in pedestrian classification, the recall and mAP increased by 5.9% and 8.5%, respectively, and in cyclist classification, the recall and mAP were also greatly improved. This is due to the fact that the edge features of the obstacle in the image are also retained well after GCANet defogging, and can be reasonably matched via the training of edge and convolution feature fusion, thus further improving the detection performance of driving obstacles in foggy weather.

## 6. Discussion

To facilitate autonomous driving in different weather conditions, and to ensure the environmental perception safety of autonomous driving, it is necessary to improve the accuracy and efficiency of obstacle detection based on visual sensors in adverse weather, especially in foggy environments. In view of the above-mentioned problems, considering that the defogging algorithm should match reasonably with the target detection algorithm, this paper has used GCANet to defog, and used the edge features retained after GCANet defogging combined with the detection method of edge and convolution feature fusion training to carry out obstacle detection, which further improves the accuracy and real-time performance of driving obstacles detection.

Different from the detection method based on conventional training after defogging, the method proposed in this paper makes full use of the edge features of the target, and reasonably matches the defogging algorithm with the detection method. Its ultimate purpose is to improve both the accuracy and time efficiency of detection. So, in the final stage, this paper comprehensively compares the two detection methods as regards accuracy and time efficiency. The image defogging algorithms used for comparison include traditional dark channel defogging [5], deep learning-based AOD-Net [7] and GCANet [16]. Dark channel defogging algorithms [5] are the most common defogging methods, while AOD-Net [7] and GCANet [16] have emerged as better methods for removing fog in recent years. These three defogging methods are used to defog the images in the foggy image test set, and the defogging images are used to test the conventional training model and the edge and convolution feature fusion training model. The comparison results are listed in Table 4.

As can be seen from Table 4, both the conventional training model and feature fusion training model require only milliseconds for single-image detection, and can thus meet the demands of real-time detection in automatic driving. However, in terms of detection accuracy, the detection method of edge and convolution feature fusion training shows greater improvements than the detection method of conventional training after defogging, which undoubtedly guarantees the safety of automatic driving. As regards the image defogging method, AOD-Net can only improve mAP by 4%, while DCP, with better performance, can improve the mAP by 45%, which is a slightly lower improvement than that of GCANet, but GCANet takes less time to defog. Through comparisons, it can be seen that the accuracy of the detection model of GCANet + edge and convolution feature fusion training yields significant improvements while ensuring the timeliness of detection.

From the results of the experiment, in foggy weather, the method proposed in this paper not only ensures real-time detection, but also greatly improves the detection accuracy of obstacles. It thus meets the demands of automatic driving, and provides a guarantee for driving safety in a foggy environment.

## 7. Conclusions

To address the issue of the low detection accuracy of driving obstacles in foggy weather, a novel detection method combining GCANet defogging with edge and convolution feature fusion training has been proposed. On the basis of analyzing the underlying features of foggy day images, the differences in edge features between the foggy images and the defogging images were compared and analyzed, then the edge feature was identified as the feature that fused with the convolutional features. Lastly, the detection performance of driving obstacles in foggy weather was improved by GCANet defogging and edge and convolutional feature fusion training. Compared with the detection results of the conventional training method, this method has improved the precision, recall and mAP of obstacle detection in foggy weather by 30%, 19%, and 45%, respectively; compared with the results of the detection method that combines GCANet defogging with conventional training, this method improved the recall and mAP of obstacle detection in foggy weather by 12% and 9%, respectively. The experiment results show that the method presented in this paper improved the performance of the detection of driving obstacle in a foggy environment.

From the image feature analysis of clear and foggy days, it can be found that the edge, color, grayscale, and other features of obstacles changed significantly under the two weather conditions, meaning foggy weather has a great effect on the underlying features of the image. When analyzing the image features before and after defogging, it can be found that although texture, color and other information will be lost after defogging, relatively intact edge features can still be retained. Therefore, combining edge features to train a detection network can improve the detection performance of the model after defogging.

Differently from the conventional detection method after image defogging, the innovation of this paper lies in that the edge features retained after defogging with GCANet were noticed and fully utilized, and the edge features and convolutional features of obstacles were fused while training the network parameters. In addition, the reasonable matching of the defogging algorithm and detection algorithm was fully considered; therefore, we can better meet the requirements for high precision in obstacle detection during automatic driving in foggy weather, while ensuring real-time performance.

However, although the method in this paper has achieved a good performance in detecting driving obstacles in foggy images, the experimental results in other adverse weather (such as rainy days) need to be verified. In addition, the precision of the proposed method is lower than that of the DCP + edge and convolutional feature fusion training method. Therefore, in the next step, we will verify the effect of this method in rainy weather and determine how it can be further improved, as well as improving the precision index of the proposed method.

## Figures and Tables

**Table 1:** The profits and drawbacks of the underlying features.

The Underlying Features of the Image	Profits	Drawbacks
Edge features	Reflects the contour and location information of obstacles	Susceptible to light intensity; will be covered in foggy days.
The grayscale features	High computational efficiency	Poor noise immunity; will be covered in foggy days
The color features	Reflects the color distribution of obstacles	Will be covered in foggy days

**Table 2:** The number of pictures and the number of obstacles in the experimental test set.

The Training Method of the Model	Number of Pictures	Detection Target	Quantity
Conventional training (KITTI data only)/GCANet + training with edge images/GCANet + conventional training/GCANet + edge and convolution feature fusion training	749	Car	3275
		Pedestrian	433
		Cyclist	142
		Total	3850

**Table 3:** Comparison of the obstacle detection performance of four model training methods applied to a foggy image test set.

The Training Method of the Model	Number of Pictures	Detection Target	Quantity	Precision	Recall	mAP@0.5
Conventional training based only on KITTI data	749	Car	3275	0.822	0.642	0.679
		Pedestrian	433	0.568	0.307	0.292
		Cyclist	142	0.486	0.5	0.451
		Total	3850	0.625	0.483	0.474
GCANet + edge images training	749	Car	3275	0.858	0.697	0.762
		Pedestrian	433	0.585	0.443	0.473
		Cyclist	142	0.634	0.493	0.496
		Total	3850	0.692	0.544	0.577
GCANet + conventional training	749	Car	3275	0.954	0.904	0.955
		Pedestrian	433	0.898	0.711	0.779
		Cyclist	142	0.954	0.669	0.762
		Total	3850	0.935	0.761	0.832
GCANet + edge and convolution feature fusion training	749	Car	3275	0.954	0.943	0.977
		Pedestrian	433	0.898	0.77	0.864
		Cyclist	142	0.929	0.93	0.942
		Total	3850	0.927	0.881	0.928

**Table 4:** Comparison of obstacle detection performance of four training methods applied to the foggy image test set.

Models	Single-Image Defogging Time (s)	Single-Image Detection Time (s)	Precision	Recall	mAP@0.5
Conventional training	—	0.008~0.023	0.625	0.683	0.474
DCP + Conventional training	0.11~0.22	0.008~0.023	0.844	0.468	0.529
DCP + Edge and convolution feature fusion training	0.11~0.22	0.008~0.023	0.936	0.879	0.925
AOD-Net + Conventional training	0.06~0.10	0.008~0.023	0.661	0.275	0.28
AOD-Net + Edge and convolution feature fusion training	0.06~0.10	0.008~0.023	0.648	0.532	0.517
GCA Net + Conventional training	0.10~0.11	0.008~0.023	0.935	0.761	0.835
GCA Net + Edge and convolution feature fusion training	0.10~0.11	0.008~0.023	0.927	0.881	0.928

## Data Availability

The raw data supporting the conclusions of this article will be made available by the authors, without undue reservation.
